# Atomistic Electrocatalysts for Modulating the Oxygen Reduction Reaction Selectivity in Carbon-Based Materials: Active-Site Engineering, Local Environment, and Magnetism

**DOI:** 10.1021/acsmaterialsau.4c00166

**Published:** 2025-01-31

**Authors:** Jose Manuel Romo-Herrera, Jonathan Guerrero-Sanchez

**Affiliations:** † Centro de Nanociencias y Nanotecnología, Universidad Nacional Autónoma de México, Ensenada B.C. 22860, México

**Keywords:** Oxygen Reduction Reaction, Carbon Matrix, Active-sites, Electronegativity, Single-atom, Transition Metals, Spin-Selection

## Abstract

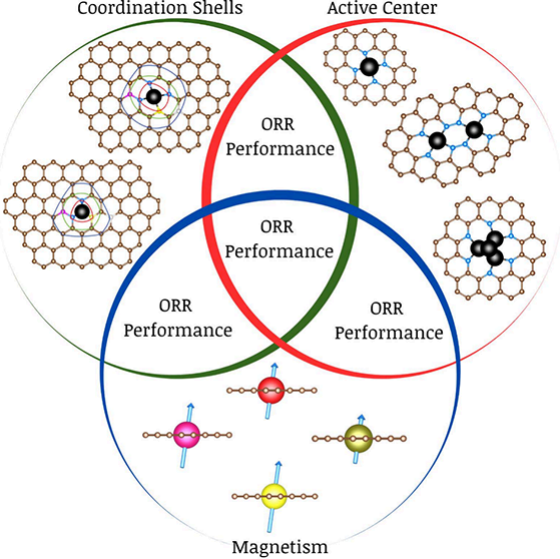

The oxygen reduction reaction (ORR) is an electrochemical process that is key to tackling global concerns regarding the conversion and storage of clean energy as well as the development of sustainable water treatment. We mainly focus on nonprecious metal catalysts, specifically harnessing Carbon-based electrocatalysts. In the current invited perspective, we highlight three main ways to control the ORR selectivity, which is still a challenge under development: (i) engineering the active sites where the use of single-atom, double-atom, or small clusters of atoms of transition metals in the carbon matrix allow including more active sites for the reaction, (ii) using coordination shells and modifying the local environment of the active-sites with more electronegative elements generates a strong positive electrostatic potential in the active site thus improving the metal–O_2_ interaction, and (iii) using spin-selection with magnetic single atoms where the magnetic moment strength of the single-atom and the triplet-to-singlet transition in the O_2_ after adsorption. More attention should be paid to this effect since the magnetic properties are directly correlated with the O_2_ adsorption strength, and at the same time, the selectivity of the O_2_ adsorption is directly related to the two- or four-electron pathway. Selectivity is commonly discussed in carbon-based catalysts but is not always linked to atomistic effects. Therefore, it is necessary to understand and rationally design alternative electrocatalysts that can synergistically combine active transition metal centers, different local environments in their coordination shells, and magnetic control.

## Introduction

The oxygen reduction reaction (ORR) is an electrochemical process that is key to renewable energy applications. Current global concerns involve converting and storing clean energy and developing sustainable water remediation processes. Electrochemical processes such as ORR can play a pivotal role in these efforts. Specifically, the ORR offers alternatives to address these concerns by either the four-electron pathway in the energy sector to convert chemical fuels into electricity by fuel cell devices^1−3^ and storing energy in metal–air batteries^3^ or the two-electron pathway in the environmental remediation sector to improve the quality of reclaimed wastewaters by on-site hydrogen peroxide generation^4−6^ for advanced oxidation processes (AOPs).

The ORR is an electrochemistry reaction in which the oxygen molecule (O_2_) interacts with electrons and hydrogens aided by an electrocatalyst material, reducing the molecule of the O_2_ into either two water (H_2_O) molecules (known as the four-electron pathway) or into a hydrogen peroxide (H_2_O_2_) molecule (known as the two-electron pathway).

The following equations summarize the main steps in the ORR pathways in an acidic medium. The *associative* mechanism for the four-electron pathway goes as follows:
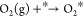
1a
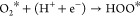
1b
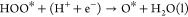
1c
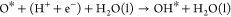
1d
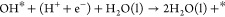
1eThe *dissociative* mechanism for the four-electron pathway goes as follows:
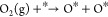
2a
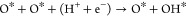
2b
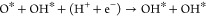
2c
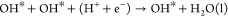
2d
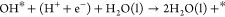
2eThe mechanism for the two-electron pathway goes as follows:
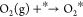
3a
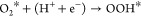
3b
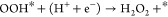
3c

**Figure 1:**
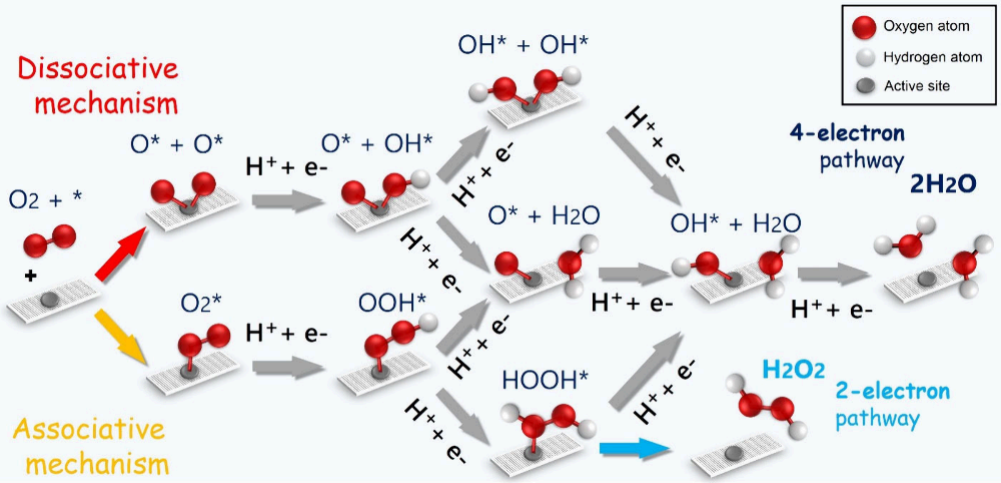
Schematic illustration of the ORR by the dissociative mechanism or associative mechanism. It should be remarked that the key role of the first step, the adsorption of the O_2_ molecule onto the catalyst active site, whose interaction strength is of paramount importance either by achieving the O–O bond cleavage or to adsorb and fix the full O_2_ molecule.

The first general step in the ORR corresponds to the adsorption of the O_2_ molecule onto the electrocatalyst site, as illustrated in Figure 1. This can result in the breakage of the O_2_ molecule bond (O^*^ + O^*^) or the adsorption of the whole molecule (O_2_^*^). This first choice will dictate a *dissociative mechanism* or *associative mechanism*. The first mechanism should guarantee avoiding the H_2_O_2_ generation (see Figure 1), making it more feasible to reach the production of two water molecules (four-electron pathway). The associative mechanism considers that the adsorbed O_2_^*^ evolves into OOH^*^, and then it could turn into O^*^ + H_2_O or H_2_O_2_^*^ (see Figure 1); for the O^*^ + H_2_O case, the O–O bond has been broken and it could end in the four-electron pathway, but for the H_2_O_2_^*^ case, there is still the possibility of breaking down the H_2_O_2_^*^ when interacting with the third hydrogen to generate chemisorbed OH^*^ and H_2_O and once more end in the four-electron pathway. However, in the last case for H_2_O_2_^*^, when the interaction is weak enough with the active site, it can be released, generating a hydrogen peroxide molecule (two-electron pathway).

Even when in both mechanisms (dissociative or associative), there are probabilities to end up generating H_2_O molecules by the four-electron pathway; an important point to remark on is the strength of the interaction of the adsorbed molecules or intermediaries with the active site since it can be identified as a way to control the selectivity of the reaction.^7^ When the electrocatalyst active site strongly interacts with the O_2_ molecule, it will avoid the two-electron pathway, fulfilling the first of two necessary conditions to end up in a successful four-electron pathway by either an associative or a dissociative mechanism. On the other hand, if the active site-molecule interaction is weak, then it can be expected to end up with the two-electron pathway.

Choosing the selectivity of the ORR represents a milestone of paramount importance, which could allow not only to choose between two different reaction pathways but also to design efficient electrocatalyst materials for crucial applications like clean energy devices or water remediation processes.

## Discussion

### ORR Selectivity: Tools for Exploring and Understanding the Mechanisms

Beyond the current efforts on the ORR using Precious Metals^8−11^ and due to their increasing costs and scarcity, searching for alternative electrocatalysts based on Non-Precious Metals is of paramount importance. The need to understand the selectivity mechanisms for the ORR to contribute to the careful control of the branching between the 2-electron and 4-electron pathways is a priority, as a key piece for designing those alternative electrocatalyst materials efficiently to apply them in clean energy devices or water remediation processes.

The optimization of the catalyst surface coverage may be explored by different means, depending on the synthesis method being used; for example, parameters of pressure, temperature, or concentration proportion of the precursors could be some of the parameters to start paying attention to or try to control the reactive sites’ density. Once a set of samples with different surface coverages or reactive site densities is achieved. Alternative characterization and evaluation techniques could be explored to analyze the structure, composition, or activity of the different samples. To analyze the reactive site density at a high-resolution level, STM could be an option. Including scanning electrochemical microscopy (SECM) should give activity data, and high-resolution transmission electron microscopy with high-angle annular dark field (HAADF) is useful to identify the metallic sites at the atomic resolution.

Computational calculations correspond to an invaluable tool for studying the ORR selectivity. A conventional approach is based on calculating the binding energies of the reaction intermediates by electronic structure calculations with the density functional theory (DFT) approximation and correlating them with the electrocatalytic activity by the concept of limiting potential (the applied electrode potential to reach thermoneutrality or downhill behavior in free energies for all of the elementary steps of the reaction). A useful way to evidence the selectivity is by constructing a *volcano* plot, which can evidence the favored pathway via the analysis of the relationship between the limiting potentials for the four-electron and two-electron ORR pathway in dependence of the OH^*^ or OOH^*^ binding energies.^12,13^ The apex of the plot is related to the best electrocatalyst performance. Farther from the apex, the catalysts could also be active for the ORR, although not as efficient as the catalysts at the apex. The volcano legs also talk about four-electron and two-electron competition; if they coincide in the same limiting potential for several binding energies, then there will be no selectivity, but if the volcano legs for the two different reaction pathways are shifted from each other, then there may be a well-defined selectivity. Recently, it has been demonstrated that slopes in the volcano legs may change by considering different mechanistic pathways related to the OOH* intermediate and by plotting it as a function of a potential dependent activity measure that depends on the overpotential and kinetic effects when evaluating the free energies.^14^ The fact that slopes can change may suggest a different way of controlling the ORR selectivity. Moreover, the volcano plot analyses have been little explored using the dependence with the O_2_^*^ binding energies as one of the initial intermediates of the ORR. This could be an intriguing parameter to be considered since, as it has been demonstrated, an initial weak interaction by a physisorption of the O_2_ molecule with the active site tends to dictate a 2-electron pathway. In contrast, a chemisorption with a stronger interaction avoids the H_2_O_2_ generation and it can lead to two H_2_O molecules generation (4-electron pathway).^7^

On the experimental side, measuring the parameters related to the selectivity of the ORR is essential. This requires robust methods for quantifying the ORR selectivity. Scanning electrochemical microscopy (SECM)^15,16^ could become an extremely useful tool to analyze in detail the ORR selectivity for different electrocatalyst materials that can be used together with the rotating ring-disk electrode (RRDE). A recent case corresponds to the analysis of the Fe-N-MWCNT electrocatalysts,^17^ where RRDE measurements initially suggested selectivity toward H_2_O (∼3.2 electrons transferred) with high loadings of the electrocatalyst for the analysis. In contrast, when ultralow loadings (accounted by meticulous atomic force microscopy (AFM)) were deposited via a spray technique on a flat boron-doped diamond substrate and analyzed using SECM, around 2.6 transferred electrons were measured, revealing a higher H_2_O_2_ production.^17^ When comparing the analysis by both techniques, they showed that more H_2_O_2_ was produced in the sample, which could have been hidden at higher loading by H_2_O_2_ entrapment by larger agglomerates.^17^ Measuring with both techniques yielded strong proof for finding the precise selectivity of the electrocatalyst. When focusing on the RRDE technique, an established method for quantifying the ORR selectivity, one should consider that canonical RRDE analysis requires that the H_2_O_2_ oxidation kinetics at the ring is fast enough to achieve that the rate of H_2_O_2_ oxidation is limited by mass transport (fast mass-transport-limited conditions). This condition is well obtained for most aqueous electrolytes; however, careful attention must be taken in nonaqueous media since sluggish H_2_O_2_ oxidation kinetics at the ring could lead to inefficient H_2_O_2_ collection and an underestimation of H_2_O_2_ selectivity.^18^ Recently, it has been shown how conventional Pt ring electrodes of RRDE in nonaqueous media do not achieve the mass-transport-limited conditions with H_2_O_2_ oxidation kinetics since H_2_O_2_ oxidation kinetics results depend on the electrolyte and solvent.^18^ They found that Au behaves better as a catalyst for nonaqueous H_2_O_2_ oxidation. They demonstrate how high-surface-area Au rings (obtained by roughening a commercial Au ring electrode) can achieve mass-transport-limited H_2_O_2_ oxidation under certain electrolytes. The methodology was applied for measuring the ORR selectivity of Fe(III)-octaethylporphyrin-chloride (Fe(OEP)Cl), obtaining 27% H_2_O_2_ selectivity. In contrast, when using the conventional RRDE with a Pt ring, the measurement gave a 7% H_2_O_2_ selectivity.^18^ This highlights how, although RRDE is an indispensable tool for measuring selectivity in ORR, careful attention must be paid when measuring ORR selectivity in nonaqueous media, which is emerging as an important medium for future ORR applications.

### ORR Selectivity Modulation: Digging into Atomistic Materials

Designing alternative electrocatalyst materials for the ORR combining characteristics such as high electronic conductivity, low cost, and made from Earth-abundant precursors, strong tolerance to harsh media (acidic or alkaline), and good activity corresponds to current challenges under development.

Metal-free carbon nanostructures doped with nitrogen atoms have progressed into a plausible option based on the local charge redistribution around the nitrogen atoms incorporated into the carbon skeleton, breaking the electroneutrality of the carbon matrix and, therefore, becoming an electrocatalyst active center. Strong effort has been focused on understanding the ORR selectivity among the different nitrogen species (i.e., pyridinic N, pyrrolic N, or graphitic N) commonly present in nitrogen-doped (N-doped) carbons. Nonetheless, the difficulty in preparing N-doped carbon samples with a single type of nitrogen species has made it difficult. The first approach was the rational preparation of model samples (pyridinic N-dominated HOPG and graphitic N-dominated HOPG), showing that the current density and onset potential increased with the pyridinic N concentration.^19^ More recently, using DFT calculations and the preparation of a set of samples of N-doped carbon nanotubes (N-CNTs) with a systematic modification of the nitrogen species proportion present, it was shown that graphitic N sites favor the two-electron pathway while the pyridinic sites with one or two pyridinic N atoms (the graphitic carbon matrix with an in-body single vacancy generates three atomic sites around the single vacancy) lead to the four-electron pathway.^7^ In this case, the selectivity of the sample can be modulated by thermal treatments, harnessing the lower thermal stability of pyridinic N and then enriching the N-CNTs samples with graphitic N.^7,20^ The selectivity is linked to the strength of the interaction between the O_2_ molecule and the active site. A higher level of complexity has been explored for the metal-free carbons when codoping with more than one type of heteroatom. The preparation of a three-dimensional (3D) ultrathin carbon nanosheet doped by nitrogen and phosphorus presented an excellent ORR performance.^21^ DFT analyses unveiled that P atoms around C atoms adjacent to graphitic N increased the charge and spin density of those C atoms. In contrast, an opposite trend resulted for the C atoms adjacent to a pyridinic N when P atoms are around.^21^

The incorporation of transition metals (TMs) as single-atom catalysts (SACs) has emerged as a cost-effective option with the utmost efficiency by the amount of material required, where N-doped carbon nanostructures are becoming essential as the substrates to anchor and stabilize the TMs single atoms homogeneously, offering great electrical conductivity at the same time. Moreover, several interesting effects appear when first-row magnetic TMs are supported on these structures. The ORR mechanism occurring on SACs based on several TMs (Fe, Co, Ni, Cu, Cr, and Mn) has been analyzed by DFT calculations using the graphene in-plane divacancy model surrounded by four pyridinic N as the TM atom anchoring site (TM-N_4_V_2_ model).^22^ The adsorption strength of the O_2_ molecule on the active site (TM-N_4_V_2_; TM = Cr, Mn, Fe or Co) correlates positively with the magnetic moments of the active species, generating stronger chemisorption as the magnetic moment increases; thus, the O_2_ ends up reduced into two H_2_O molecules (four-electron pathway). On the other side, physisorption of the O_2_ molecule on TM-N_4_V_2_ (TM = Ni or Cu), a nonmagnetic and magnetism-induced system, respectively, ends up with an H_2_O_2_ molecule through the two-electron pathway.^22^ Interestingly, the composition of the TM-SACs and their associated magnetic moments could represent key parameters to consider when designing electrocatalysts with a chosen reaction selectivity (ORR pathway) and application. The electrocatalytic activity of SACs for the ORR four-electron pathway has caught most of the attention; nonetheless, in recent years, the two-electron pathway has gained attention due to its importance for the direct on-site production of H_2_O_2_ for the chemical industry and improved reclaimed wastewater quality processes. A critical point in increasing the ORR selectivity for the two-electron pathway is to minimize the O–O bond of the adsorbed O_2_ molecule. The influence of the TM on the catalytic activity and selectivity toward H_2_O_2_ production of SACs electrocatalysts has been analyzed in detail.^23,24^ The effect of the nature of 3d metal on the electrocatalytic selectivity for ORR was studied with a series of TM SACs (TM = Mn, Fe, Co, Ni, and Cu) using RRDE and rotating disk electrode (RDE) in acidic media, together with DFT calculations.^23^ The ORR two-electron pathway involves a single adsorbed HOO^*^ intermediate, making the binding free energy of HOO^*^ a critical parameter. However, the existence of a constant scaling between the binding free energy of HOO^*^ and HO^*^, made possible the usage of the binding free energy of HO^*^ as a descriptor for the two-electron ORR, which allows the analysis of volcano plots for both the two-electron and the four-electron ORR under the same descriptor for the HO^*^ intermediate. When the activity of the different TM SACs is compared, it is shown how the Fe and Mn SACs sites cause a strong binding of HO^*^, resulting in the predominance of a four-electron pathway over the two-electron pathway. Nonetheless, the Co SACs present an optimum binding free energy for HO^*^, placing the Co SACs close to the top of the two-electron volcano. They prepared the series of samples experimentally and, when measuring their electrocatalytic activity and selectivity, demonstrated that the Co SACs sites exhibited the highest ORR selectivity toward the H_2_O_2_ production.^23^ Similarly, it was theoretically designed by predicting the activity volcano relation and experimentally demonstrated that Co SACs in N-doped graphene had the optimum adsorption energy for the OOH^*^ intermediate, exhibiting a high H_2_O_2_ production rate in an acidic medium among a group of TM SACs (TM = Mn, Fe, Co, Ni, and Cu). It is shown how the Co SACs sites possess the optimal d-band centers for the selectivity toward the ORR two-electron pathway. It is described that the dependence on the electronic interaction of the intermediates with the TM atom is the main relation for the ORR activity. It is shown how the binding energies of the intermediates (OOH^*^, O^*^, and OH^*^) scale with the number of valence electrons in the TM atom from Mn to Cu, being weaker the binding of these intermediates with a larger number of valence electrons and therefore strongly related to the d-band center of the TM atom. Therefore, the ORR on the Ni and Cu SACs shows selectivity for the two-electron pathway but with a large overpotential due to the large OOH^*^ reduction barrier; the ORR on the Mn and Fe SACs presents selectivity for the four-electron pathway due to a so strong binding of O_2_ making favorable in free energy the OOH^*^ reduction to O^*^; while the Co SACs presents an optimal d-band center with an OOH^*^ free energy neither too strong nor too weak, positioning it nearly at the top vertex of the volcano plot for the two-electron pathway, suggesting its high activity for the H_2_O_2_ production. Besides, the higher barrier for OOH^*^ reduction to the O^*^ intermediate on the Co SACs, compared with those of Mn and Fe SACs, should enhance the selectivity of the Co SACs to H_2_O_2_.^24^

The temperature effect (25–80 °C) on the ORR selectivity has also been studied on iron- and nitrogen-doped carbon (Fe–N–C) catalysts in acidic media.^25^ It is shown that the H_2_O_2_ yield increases slightly with the temperature, which is attributed to the fact that the thermodynamic overpotentials for the two competing reaction pathways shift differently. Moreover, by analyzing the performance of the Fe–N–C catalyst during prolonged high-temperature exposure at ORR conditions by sequential heating/cooling experiments, they observed an increase in selectivity for the 2-electron pathway after several experiments, an irreversible increment due to a different effect caused by catalyst degradation.^25^ This shows the importance of considering temperature effects on the ORR selectivity.

**Figure 2:**
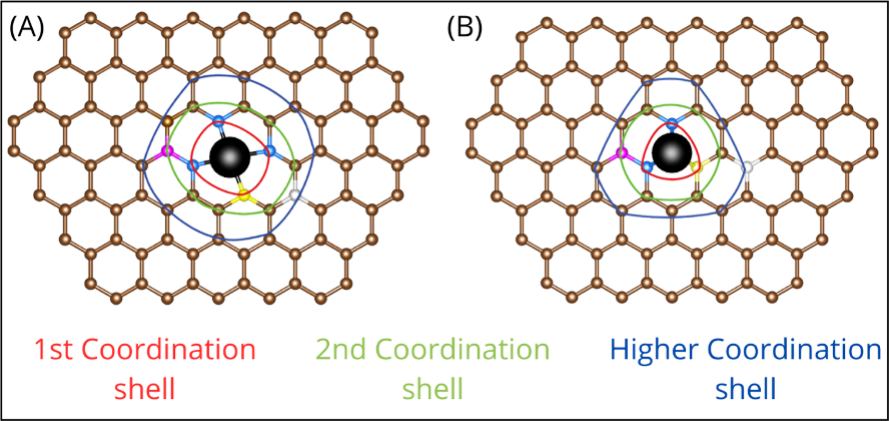
(A) Coordination shells for engineering the ORR performance of a 4-fold coordinated metal atom site. (B) Coordination shells related to a 3-fold coordinated metal atom site. Blue atoms represent the commonly used N atoms, while yellow, pink, and white represent different atomic species with different electronegativity such as B, P or S.

Advances have also been made in modifying the local coordination environment of SACs to improve the electrocatalytic reactions like N_2_RR^26^ and ORR. Modifications can be performed in the first, second, or higher coordination shells, as depicted in Figure 2. In coordination chemistry and crystal field, atoms coordinated to the active metal center significantly affect the catalytic site since metal active centers can be 4-fold (similar performance to Pt catalysts) or 3-fold coordinated (when decreased size sites are available producing metal exposure), which represent a different approach to modify/optimize their electronic structure.^27^ Modifying such a local environment induces a change in properties and may enhance the energetics of the reaction intermediates.^27^

For carbon-based SACs, the first coordination shell usually possesses four pyridinic N atoms (Figure 2A). Modifying one or more of these N atoms in the first coordination shell can completely change the ORR performance. The common already used atoms are S, O, B, and P, in which the combination of lone pairs and different electronegativity helps to tune the ORR performance in each case, mainly toward the four-electron pathway as described in detail in ref (27). Modifications in the second and higher coordination shells can also help to improve the catalysts’ performance. Usually, species with different electronegativity are used to generate a strong positive potential in the atomic active center, which helps strengthen the metal–O_2_ interaction, thus controlling the ORR selectivity.^27^

A different approach to modifying the local environment is based on the size of the anchoring site. This can be done by using three N atoms around a single vacancy as the anchoring site for the metal active center (Figure 2B). This type of decreased size site modifies the arrangement of the atoms in the site, causing the metallic atom to remain outside of the graphitic sheet, protruding of the plane, and generating a highly localized and positive potential that strongly attracts O_2_ molecules. It can even change the selectivity of some TM SACs.^28^ Cu and Ni SACs in a 4-fold coordination site (Figure 2A) proceed via a two-electron pathway, nonetheless, in a 3-fold coordinated site (Figure 2B), both metals change selectivity toward the four-electron pathway.^28^ To the best of our knowledge, this type of decreased-size site with a 3-fold coordination environment has not been explored with modifications in the first, second, or higher-order coordination shell modifications. Such modifications could be beneficial for the engineering of novel electrocatalysts, generating even more positive potential metal centers, which should affect the metal–O_2_ interaction as a key parameter for the ORR.

**Figure 3:**
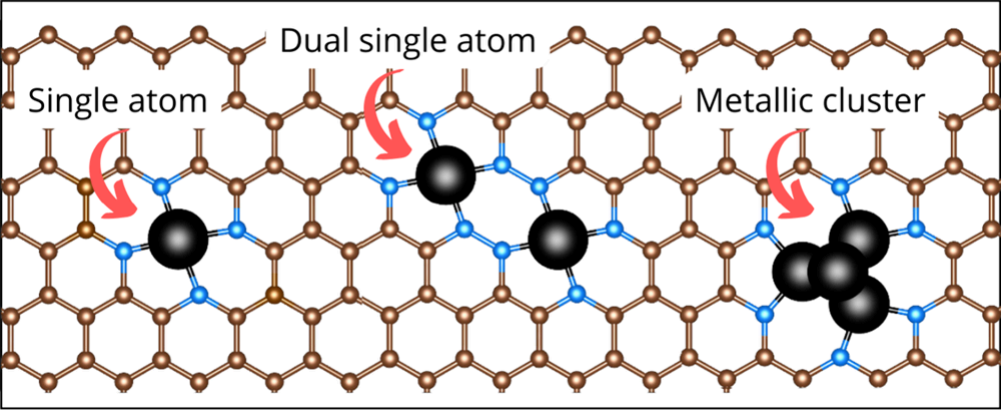
Schematic illustration of different metallic centers and their first coordination shell. From left to right: single atom site, dual single atom site, and metallic cluster site models.

A different approach explored has been the modification of the metallic centers rather than their surroundings,^29−32^ as schematized in Figure 3. Engineering the electrocatalysts by incorporating dual single metallic atoms is another way to generate strong metallic centers–O_2_ adsorption interactions, which can result in modification/optimization of the ORR selectivity. The key parameter in this kind of arrangement relies on the metallic center distance since it needs to be short enough to trap the O_2_ molecule in bridge-like sites.^33^ For example, Fe–Zn pairs on carbon substrates enhance ORR performance in acidic conditions for Fe–Zn distances of around ∼3 Å.^33^

Small metallic clusters, composed of three, four, or five metallic atoms, can represent alternative electrocatalyst sites that combine the atomic environment with the localized spatial arrangement, giving them a favorable surface topology. The cluster size is the critical parameter since it needs to be controlled to harness the advantage of atomic confinement and a homogeneous activity. An approach to achieve this could be by engineering small clusters that emerge from a certain amount of single or dual atom sites as the seed site and then complete the cluster on top of these atom’s arrangement. This is an unexplored field, and very few reports have been published about this kind of design. A recent work reports cobalt atom clusters coexisting together with single cobalt atom sites dispersed on N-doped carbon substrates to enhance the ORR activity and preserve its stability in seawater as the electrolyte.^34^ This work describes how the cobalt clusters preferentially trap the Cl^–^ anions due to their positive potential, helping to keep the Co single atoms sites fully exposed to carry out the ORR.^34^ Studies remain to be done on how small clusters and their different electrostatic potentials, influenced by their local environment, can perform in the ORR.

### ORR Selectivity: On the Role of Magnetism

Although transition metals have emerged as efficient alternatives to obtain SACs for electrochemical reactions, not many studies have elucidated the role of their intrinsic magnetism in these reactions’ activity and selectivity, the role of magnetism remaining underexplored in several catalysts. Efforts have been made to study high-spin state single-atoms into defective BN nanosheets for the nitrogen reduction reaction, where the sites with higher spin magnetic moment interact strongly with N_2_, then reducing the limiting potentials to carry out the reaction.^35^ Although the activity is related to the metallicity of the catalyst. Still, when using transition metals, another degree of freedom should be considered in these systems: the electron spin, especially in cases that involve spin-triplet O_2_.^36^ This section describes some of the recent investigations using magnetic TM single-atom catalysts.

Recent computational studies have evidenced the appearance of spin selection in porous g-C_3_N_4_/CeO_2_(111) heterostructures where Mn atoms are intercalated between the heterostructure. Through the g-C_3_N_4_ pores, Mn strongly interacts with the O_2,_ weakening its double bond and involving a triplet (2 μ_B_)-to-singlet (∼0 μ_B_) transition.^36^ In electrocatalysis, the catalysts must follow the Sabatier principle, which states that adsorption strength should be equilibrated, not be too weak or too strong, to reach maximum activity. Then, to determine the ORR selectivity, we must focus on the full reaction step-by-step to locate the limiting step, which separates the four- and two electron pathways. Very recently, it has been observed that 4-fold pyridinic N atoms with a Fe single-atom site in a carbon-based system can be readily improved by incorporating an axial S atom in the first coordination shell and a P atom in the second one, which induces an electronic rearrangement and consequently a spin polarization improvement.^37^ Also, the increased coordination in Fe may induce a relocation of the d-band center (local modification of its electrostatic potential), modifying the interaction with O_2_.^37^ In this computational investigation, a system in which the Fe coordination was changed (from 4 to 5 bonds), adsorbing S directly on Fe, allowed the shift of the d-band center farther from the Fermi level to negative energies, thus reducing the adsorption energy of the intermediate ^*^OH + H_2_O + H^+^ + e^–^ and enhancing the ORR activity.^37^ The reduction in adsorption energy obeys the principle in which d-states are now shared with five atoms (not four as in the original N pyridinic system), leaving less available states to share with the reaction intermediate.

**Figure 4:**
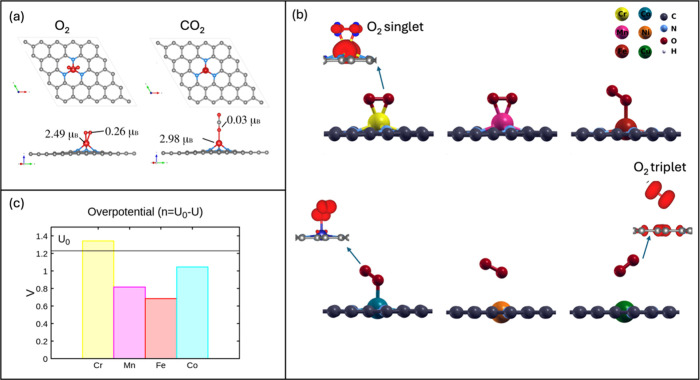
(a) Proof of concept for adsorption of O2 and CO2 on the magnetic single atom Fe incorporated into the three pyridinic nitrogen graphene system (TM-N_3_V_1_), (b) adsorption of O_2_ on several magnetic and nonmagnetic single atom systems incorporated into the four pyridinic nitrogen graphene system (TM-N_4_V_2_) and spin density isosurfaces showing the O_2_ evolution in different adsorption configurations, (c) overpotential reported for the ORR reaction on Cr-N_4_V_2_, Mn-N_4_V_2_, Fe-N_4_V_2_, and Co-N_4_V_2_ systems. Panel (a) has been adapted from Figure 5(a) and 5(d) in ref (28) with permission from *ACS Appl. Energy Mater.***2024**, *7*, 4794–4802. Copyright 2024, American Chemical Society. Panels (b) and (c) have been adapted from Figures 2(a), 3(e), and S2 in references (22 and 28) with permission from *ACS Appl. Nano Mater.***2024**, *7*, 338–347 and *ACS Appl. Energy Mater.***2024**, *7*, 4794–4802. Copyright 2024, American Chemical Society.

Interestingly, in this investigation, the authors did not discuss selectivity in the ORR, although the concept of spin selection is behind the first ORR step. Once the coordination in Fe gets modified, there is a slight enhancement in the Fe magnetic moment from 1.978 μ_B_ to 2.064 μ_B_, which suggests strong side-on adsorption of the O_2_ molecule, and then the reaction proceeds in the associative pathway (see Figure 1 and eq 1) for generating the four electron ORR reaction. The free energy diagram of the full ORR is also studied at *U* = 0 V, from which the reaction steps are spontaneous and exothermic.^37^ Another hypothesis in the S-modified system is that the S bonds may induce higher Fe valence states and electron delocalization around the active Fe center, which could drive a better performance of the ORR reaction.^38^ These recent reports show emerging systems incorporating S into the first coordination shell to improve the ORR. However, more quantum mechanical calculations are needed in transition metal single-atom carbon-based systems to reveal the connection between magnetism and selectivity. Spin is mentioned, but they still did not consider it when interacting with the O_2_ molecule to see if spin selection exists. We proposed a proof of concept to elucidate this fact in ref (28) as summarized in Figure 4(a) in which we used a model containing a single vacancy with three pyridinic N atoms as the TM atom anchoring site (TM-N_3_V_1_). We compare the adsorption energies of the adsorption energy of the O_2_ and the adsorption energy of the CO_2_ when interacting with the Fe active site. O_2_ has an adsorption energy of −3.11 eV/O_2_ molecule, while it has a CO_2_ adsorption energy of −0.39 eV/CO_2_ molecule. In the first case, O_2_ experiences a triplet-to-singlet transition after interacting with the Fe atom (Figure 4(a)); the spin selection observed in this system and electrostatic interactions are the main reasons for the large adsorption energy. In the case of the nonmagnetic CO_2_ molecule, the interaction is just electrostatic.

Recently, we have carried out a computational study using the spin selection concept linked to selectivity;^22^ in this work, we evaluated the effect of the magnetism induced in TM-N_4_V_2_, with TM = Cr, Mn, Fe, Co, Ni, and Cu, on the O_2_ adsorption and the further ORR performance. The adsorption energy has the following trend: Cr (−2.05 eV) > Mn (−1.00 eV); Fe (−0.87 eV) > Co (−0.78 eV) > Ni (−0.16 eV) > Cu (−0.12 eV). Adsorption strength is directly related to the magnetic characteristics of O_2_ and the single atom, see Table 1.

**Table 1:** Adsorption Energy, TM Magnetic Moments, TM Magnetic Moments after O_2_ Adsorption, and Magnetic Moments of the O_2_ Molecule Adsorbed on the TM^22^

System	*E*_ads_ (eV)	MM single atom, before O_2_ ads (μ_B_)	MM single atom, after O_2_ ads (μ_B_)	MM O_2_ adsorbed (μ_B_)
Cr–N_4_V_2_	–2.05	3.78	1.78	0.15
Mn–N_4_V_2_	–1.00	3.29	1.88	0.14
Fe–N_4_V_2_	–0.87	2.01	0.83	0.55
Co–N_4_V_2_	–0.78	0.92	0.02	0.83
Ni–N_4_V_2_	–0.16	0.00	0.06	1.52
Cu–N_4_V_2_	–0.12	0.52	0.53	1.49

The data in Table 1 show that as the magnetic moment of the single atom species increases, the adsorption energy is larger. For example, in the case of Cr and Mn, the O_2_ molecule experiences a triplet-to-singlet transition upon adsorption, driving the O_2_ bond weakening, Figure 4(b). When O_2_ interacts with Fe and Co, the adsorption is not as strong, driving to a side-on configuration, and the triplet-to-singlet transition is not complete (in Fe and Co cases, O_2_ is not completely demagnetized), resulting in an associative pathway of the ORR (Figure 4(b)). Finally, when the single atoms are not magnetic (Ni) or with a slightly induced magnetic moment (Cu), the O_2_ adsorption is weak, and O_2_ remains in its triplet state, see Table 1 and Figure 4(b). Such a state is modified when forming the HOO^*^ species. Then, we conclude that the first adsorption step is key to controlling the selectivity toward four or two electrons in the ORR. Weakening the O=O bonds through chemisorption helps to reach the four-electron pathway, while physisorption goes through the two-electron path (see Figure 1), which is controlled by the magnetic characteristics of the single atom. Considering the Sabatier principle, we analyzed which of the magnetic single atoms is best suited for the ORR in the four-electron path through free energy analysis.^22^ In Figure 4(c), Alvarado et al.,^22^ reported the overpotential for the ORR in the four magnetic single atoms Cr, Mn, Fe, and Co. Notice that the species with large magnetism (Cr) is not well suited for the ORR since it forms strong bonds with O and OH, generating large energy barriers for the last ORR steps. The Mn and Fe behave better due to their lowest overpotentials; see Figure 4(c). In contrast, although Co magnetism is not strong, its overpotential is large because the energy barrier to reach the first hydrogenation is large, precluding a good yield in the ORR reaction. Ni and Cu proceed toward the two-electron path since the adsorption is not strong enough to break down the O=O bonds, making it more probable to reach the HOO^*^ state; the reaction proceeds as shown in eqs 3.

**Figure 5:**
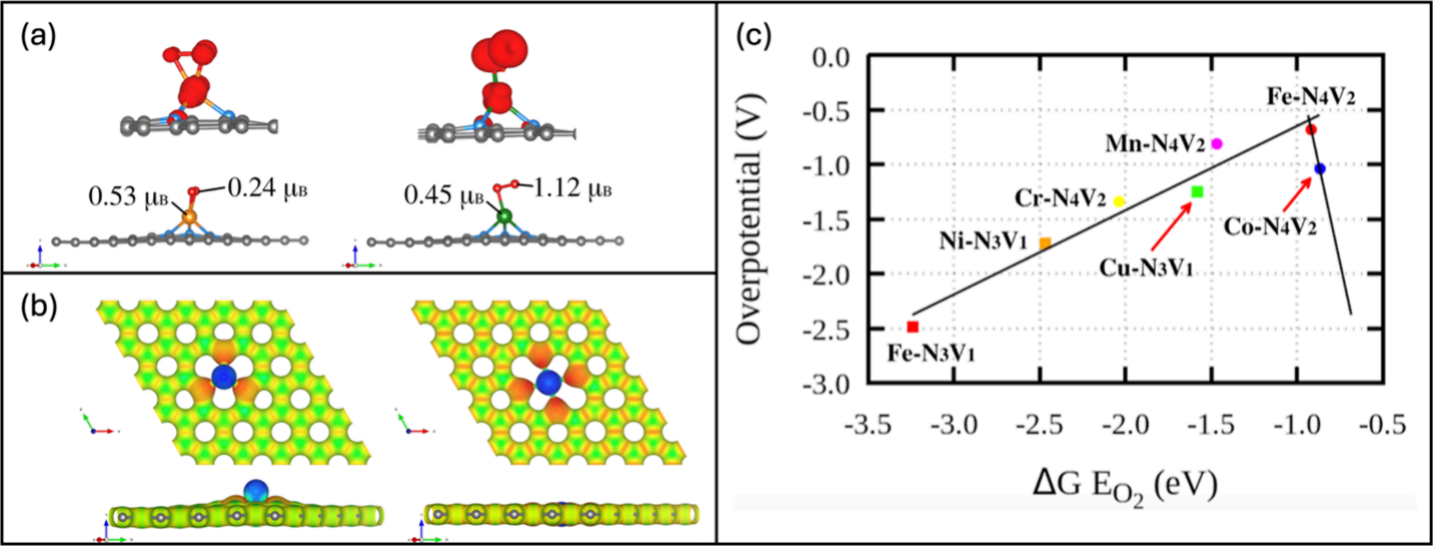
(a) Adsorption of O_2_ on several magnetic and nonmagnetic single atom systems incorporated into the three pyridinic nitrogen graphene system (TM-N_3_V_1_) and spin density isosurfaces showing the O_2_ evolution in different adsorption configurations, (b) electrostatic potential isosurfaces, left side Cu-N_3_V_1_ and right-side Cu-N_4_V_2_, (c) volcano plot comparing different TM in both TM-N_3_V_1_ and TM-N_4_V_2_ systems. Panel (a) has been adapted from Figure 5(b), 5(c), 5(e), and 5(f) in reference (28) with permission from *ACS Appl. Energy Mater.***2024**, *7*, 4794–4802. Copyright 2024, American Chemical Society. Panels (b) and (c) were reprinted from Figure 4 and Figure 5(d) in reference (28) with permission from *ACS Appl. Energy Mater.***2024**, *7*, 4794–4802. Copyright 2024, American Chemical Society.

Recently, we further explored into the role of magnetism and the single-atom local environment in the selectivity of the ORR toward the four-electron path using quantum chemical calculations. We focused on the most favorable magnetic single atom Fe and the ones that generated the two-electron pathway, Ni and Cu. The model system accounted for a different atomic environment, the TM-N_3_V_1_, which has a single vacancy with three N pyridinic sites bonded to the TM. In this model, TM shifts upward from the monolayer atoms level, generating a prominent exposure of the single atom, as seen in Figure 4(a). The first thing to observe is that the O_2_ adsorption energy in the Fe–N_3_V_1_, Ni–N_3_V_1_, and Cu–N_3_V_1_ increases due to the exposed TM atoms, see Table 2. In the TM-N_4_V_2_ model are Fe–N_4_V_2_ = −0.87 eV, Ni–N_4_V_2_ = −0.16 eV, and Cu–N_4_V_2_ = −0.12 eV, while in the TM-N_3_V_1_ model, Fe–N_3_V_1_ = −3.11 eV, Ni–N_3_V_1_ = −1.17 eV, and Cu–N_3_V_1_ = −1.45 eV, a difference of 2.24, 0.39, and 2.29 eV. The large difference in adsorption energy is related to the spin selection and to the stronger electrostatic interactions due to the exposed TM atoms. In the case of Fe, the O_2_ strongly chemisorbs, generating selectivity toward the dissociative four-electron pathway;^28^ see eqs 2 and Table 2. Figure 4a shows that O_2_ experiences a triplet-to-singlet transition upon interacting with Fe and weakening of the O=O bonds weakening. For Ni, in contrast to the planar Ni–N_4_V_2_ model in which the system was not magnetic, the Ni–N_3_V_1_ magnetizes and generates a strong interaction with the O_2_ molecule, in which the chemisorption conduces to the ORR dissociative four-electron pathway, see eqs 2(28) and Table 2. In this case, a triplet-to-singlet transition mediates the interaction; see the left side of Figure 5(a). When focusing on Cu, in the TM-N_3_V_1_ model, there is no magnetic moment; however, the O_2_ interaction happens via a side-on configuration, forming one Cu–O bond and a partial O_2_ demagnetization (right side, Figure 5(a) and Table 2). The ORR reaction proceeds via the associative four-electron pathway.^28^ This interaction is mediated mainly by the electrostatic characteristics generated due to the protruded Cu atom, as shown in the more exposed and positive electrostatic potential isosurface, left, part of Figure 5(b). Notice in the right part of the same figure that even though it is the same species (Cu), in the Cu-N_4_V_2_ model, there is no Cu protrusion, remaining in the same plane as the graphene and nitrogen atoms and making the Cu-O_2_ interaction more difficult due to the four orange repealing spots.

**Table 2:** Adsorption Energy, TM Magnetic Moments, TM Magnetic Moments after O_2_ Adsorption, and Magnetic Moments of the O_2_ Molecule Adsorbed on the TM^a^

System	*E*_ads_ (eV)	MM single atom, before O_2_ ads (μ_B_)	MM single atom, after O_2_ ads (μ_B_)	MM O_2_ adsorbed (μ_B_)
Fe-N_3_V_1_	–3.11	3.04	2.49	0.26
Ni-N_3_V_1_	–1.17	1.08	0.53	0.24
Cu-N_3_V_1_	–1.45	0.00	0.45	1.12

a Data obtained from ref (28).

From all the discussed data, we can conclude that the reaction proceeds via the dissociative four-electron pathway when there is a triplet-to-singlet transition and toward the associative two-electron pathway when partial demagnetization in the O_2_ appears. In the case of no triplet-to-singlet transition in O_2_, the weak interaction makes the ORR proceed via the two-electron pathway. The volcano plot reported in ref (28) and in Figure 5(c) confirms our assertions. First, we notice that Fe-N_4_V_2_ and Fe-N_3_V_1_ share the same species, but the overpotential for the former is still lower. This means that coordination shells, reaction site engineering, and magnetism should be tuned to fulfill the Sabatier principle and get improved and efficient electrocatalysis.

Moreover, experimental proof of chiral-induced spin selection has recently appeared in the literature on iron phthalocyanine (FePc) self-assembled to the gold electrode using chiral peptides (L and d enantiomers).^39^ The chiral peptides (NH_2_(AAK)_*n*_M (with *n* = 1 to *n* = 4 and C-terminal as amide, *n* = 1 stand for 1L or 1D depending on the handedness) optimize the O_2_ interaction with Fe by inducing spin filtering and further spin selection as a function of the chirality and length,^39^ 3D-enantiomers promotes the highest activity of the FePc for the ORR by effectively driving a triplet-to-singlet transition in the O_2_, strengthening the adsorption and producing the four-electrons pathway.^39^ Also, the ORR activity can be greatly enhanced in the 4-fold pyridinic N atoms with a Fe single-atom site in a carbon-based system by incorporating adjacent atomic-size Fe clusters. The incorporation of these adjacent clusters generates a transition in Fe from high spin Fe(III) to medium spin Fe(II), facilitating the interaction with the π* antibonding orbitals of the oxygen molecule.^40^

There is an open field in the case of the adsorption strength and selectivity in the ORR mediated by double transition metals or small clusters of transition metals. For example, if more redox sites are put together, things may change because magnetic species behave differently than a single atom. O_2_ will have more ways to interact, and selectivity is one of the first things that may change. Research must be done to determine the link between magnetism and cluster size and its effect on adsorption strength and limiting potentials. Another thing to dig into is combining elements with large magnetic moments such as Cr or Mn with others of small magnetic moments such as Ni or Co, which may bring interesting synergistic effects toward modifying the adsorption strength and ORR selectivity. Finally, subjecting the magnetic catalysts to strong magnetic fields could also be a way to tune and engineer the adsorption and selectivity of the ORR.

## Conclusions

ORR selectivity is still a challenge that should lead to alternatives for the energy conversion and storage sector (fuel cells and metal–air batteries) and the environmental remediation field by improving the quality of reclaimed wastewater.

Even though the four-electron pathway of the ORR is already a highly valued reaction in the energy sector, it is equally important to highlight the significance of the two-electron pathway for the on-site generation of H_2_O_2_, which is required in advanced oxidation processes to improve water quality.

More precise characterization tools should improve our understanding of the ORR selectivity mechanisms, highlighting scanning electrochemical microscopy as a powerful technique to be harnessed. Moreover, *in situ* and *operando* characterizations are emerging as tools to gain further insights.

As highlighted in the present perspective article, there are three main approaches to modulate the selectivity for the oxygen reduction reaction, avoiding the precious metals, with carbon-based electrocatalysts: (i) through coordination shells including different heteroatoms doping (N, P, S, among others), (ii) by modifying the reactive sites with single, double, or multiple atoms clusters using transition metals, and (iii) the control through spin selection with magnetic single atoms.

Remarkably, even when several reports have used a single atom to improve the electrocatalysts, we would expect more attention to the magnetic characteristics in the next years since these properties are directly entangled with the interaction strength of the active site-O_2_ molecule, generating strong (triplet-to-singlet transition) or weak O_2_ adsorptions as a direct way to reach the ORR selectivity either to the four- or two-electron pathway, respectively.

Specifically, it was illustrated with transition metals SACs in Carbon-based substrates,^22,27,28^ how a triplet-to-singlet transition experienced by the O_2_ molecule upon adsorption to the active site, can drive the O_2_ bond weakening, a crucial phenomenon involved in the ORR selectivity mechanisms. Nonetheless, the strength of such interaction is of paramount importance, which is directly related to the magnetic characteristics of the system. Moreover, if the single atoms are not magnetic, the O_2_ adsorption is weak, and O_2_ remains in its triplet state. Therefore, the first adsorption step is key to controlling the selectivity toward four or two electrons in the ORR.

Further work must be done to understand the hidden magnetism in the already reported electrocatalysts and its role in the ORR selectivity mechanisms. It is also necessary to rationally design alternative electrocatalyst materials that combine synergistically the best of coordination shells, active transition metal centers, and intrinsic or induced magnetism.
